# Effectively Predicting the Presence of Coronary Heart Disease Using Machine Learning Classifiers

**DOI:** 10.3390/s22197227

**Published:** 2022-09-23

**Authors:** Ch. Anwar ul Hassan, Jawaid Iqbal, Rizwana Irfan, Saddam Hussain, Abeer D. Algarni, Syed Sabir Hussain Bukhari, Nazik Alturki, Syed Sajid Ullah

**Affiliations:** 1 Department of Creative Technologies, Air University Islamabad, Islamabad 44000, Pakistan; 2 Department of Computer Science, Capital University of Science and Technology, Islamabad 44000, Pakistan; 3 Department of Computer Science, University of Jeddah, P.O. Box 123456, Jeddah 21959, Saudi Arabia; 4 School of Digital Science, Universiti Brunei Darussalam, Jalan Tungku Link, Gadong BE1410, Brunei; 5 Department of Information Technology, College of Computer and Information Sciences, Princess Nourah bint Abdulrahman University, P.O. Box 84428, Riyadh 11671, Saudi Arabia; 6 Department of Electrical Engineering, Sukkur IBA University, Sukkur 65200, Pakistan; 7 Department of Information Systems, College of Computer and Information Sciences, Princess Nourah bint Abdulrahman University, P.O. Box 84428, Riyadh 11671, Saudi Arabia; 8 Department of Information and Communication Technology, University of Agder (UiA), N-4898 Grimstad, Norway

**Keywords:** heart disease dataset, disease prediction, supervised learning, machine learning

## Abstract

Coronary heart disease is one of the major causes of deaths around the globe. Predicating a heart disease is one of the most challenging tasks in the field of clinical data analysis. Machine learning (ML) is useful in diagnostic assistance in terms of decision making and prediction on the basis of the data produced by healthcare sector globally. We have also perceived ML techniques employed in the medical field of disease prediction. In this regard, numerous research studies have been shown on heart disease prediction using an ML classifier. In this paper, we used eleven ML classifiers to identify key features, which improved the predictability of heart disease. To introduce the prediction model, various feature combinations and well-known classification algorithms were used. We achieved 95% accuracy with gradient boosted trees and multilayer perceptron in the heart disease prediction model. The Random Forest gives a better performance level in heart disease prediction, with an accuracy level of 96%.

## 1. Introduction

The healthcare sector generates a lot of data regarding patients, diseases, and diagnoses, but it is not being appropriately analyzed, so it is not providing the value it should be. Heart illness is the prime reason of death. Rendering to the World Health Organization [1], CVDs are the largest cause of mortality globally, resulting in the deaths of an estimated 17.9 million individuals each year. The healthcare industry generates a lot of data regarding patient, diseases, and diagnoses, but it is not properly analyzed, so it does not have the same impact as it should on patient health [1].

CVDs include coronary artery, rheumatic heart disease, vascular disease, and various heart and blood vessel problems. Four out of every five CVD fatalities are caused by strokes or heart attacks. Among the total deaths, one-third occurs with persons below the age of 70 [2]. Sex, smoking, age, family history, poor diet, cholesterol, physical inactivity, high blood pressure, overweightness, and alcohol use are the key risk influences for heart disease. Heart disease is also caused by hereditary risk factors such as diabetes and high blood compression [3]. Physical idleness, fatness and unhealthy diet are some of the subordinate reasons that increase the risk. Fatigue, palpitations, sweating, back pain, chest pain, shoulder and arm pain, breath shortness and overall weakness are the most common symptoms. The most recurrent sign of deficient blood stream to the heart is still chest pain. In medical terminology, this type of chest pain is known as Angina [4]. There is examination available to help diagnose the disease, such as X-rays, MRI scans, and angiography. Though, there are times when there is a shortage of resources in an emergency due to non-availability of medical apparatus. In cardiovascular disease, the time is as important as every moment of diagnosing and treating the disease is counted [4].

Cardiac midpoints and outpatient departments produce huge outlay of data regarding the diagnosis of heart diseases, and the potential demand for improvement of big data analytics regarding cardiovascular overhaul and patient consequences is vast [5]. However, due to noise, incompleteness, and irregularity, it is hard to make specific, accurate, and well-grounded decisions using the data. Nowadays, AI is playing an important role in the field of cardiology, appreciations to massive advancements in equipment, big data, knowledge storage, acquisition, and recovery [6]. Using various data mining techniques, researchers used preprocessing methods on the data to make verdicts using various ML models [7]. In the cataloguing of genetic cardiac illnesses and control subjects, a widespread set of ML algorithms with their variations is used to predict the early stages of heart failure [8,9]. KNN, DT, SVC, LR, and RF machine algorithms are examples of heart attack prediction algorithms [10]. Machine learning approaches can be divided into three categories [11]: Supervised ML: task drive, labeled data (classification/regression); Unsupervised ML: data-driven, unlabeled data (clustering); Reinforcement Learning: learning from mistakes (playing games).

In this study, supervised ML classifiers are used to show how different models can predict the existence of heart disease and compare the accuracy of these classifiers, such as Logistic Regression (LR), k-Nearest Neighbors (kNN), XGBoost (XGB), Support Vector Machine (SVM), Stochastic Gradient Boosted Tree (GBT), Naive Bayes (NB), Neural Network (NN), Decision Tree (DT), Radial Basis Function (RBF), Random Forest (RF), and Multi-Layer Perceptron (MLP). 

The rest of the paper is ordered as: Section 2 contains the literature review. The proposed methodology is discussed in Section 3. The experiment’s results are discussed in Section 4. To sum up, conclusions are inscribed in Section 5.

## 2. Materials and Methods

Research efforts are related to information exploration using ML classifiers. Several papers have been written by researchers and practitioners to predict the presence of heart disease. Numerous studies and approaches have been developed to date to classify heart disease with data mining and ML. The authors of [12] proposed a detailed review about the study on the claims of ML in the domain of heart illness. The authors proposed a dataset which possess the required samples and data that could be used to construct an efficient method for the prediction of heart diseases. Preprocessing of the dataset has to be performed efficiently for formulating the dataset which will be used by the ML algorithm, in order to produce excellent results.

The authors of the study also endorsed the use of a suitable algorithm, such as an ANN or a DT, when developing a prediction model. ANN outperformed DT in most models for predicting heart disease. In [13], the authors projected a technique for envisaging heart disease using data analytics tools and ML techniques such as ANN, DT, Fuzzy Logic, NB, kNN, and SVM. The paper also includes a performance analysis of the algorithm as well as a summary of previous research. The author of [14] proposed an architecture that includes input data preprocessing before training as well as testing on various algorithms. The use of Adaboost is recommended by the author because it improves the presentation of all ML algorithms. The idea of fine-tuning parameters to achieve high accuracy was also supported by the author.

Researchers suggested a deep learning method for analysis and diagnosing of heart illness [15] using the UCI dataset. Furthermore, they expressed that that Deep Neural Network can be crucial in enhancing the overall classification quality in the field of heart disease analysis and diagnosis. They showed that Talos Hyper parameter optimization outperforms other techniques for model optimization. KNN, RF, SVM, and DT algorithms were discussed as available ML models for the forecast of heart disease with high accuracy, recall, and precision. The classification produced using SVM gave the highest accuracy of 86% in their prediction model on the UCI ML repository for heart diseases [16].

The authors of [17], using four ML algorithms and one NN, compared performance quantities to cardiac disease detection. To predict cardiac doses, the authors evaluated the algorithms in constraints of accuracy, recall, precision, and F1 settings. The Deep NN algorithm correctly identified heart disease 98% of the time. The author of [18] focused on the algorithm’s implementation with a medicinal dataset to demonstrate its utility in early disease prediction. According to the findings of the study, boosting and bagging are powerful ensemble approaches for enhancing the estimate accuracy of classifier whose accuracy is relatively low, as their performance in predicting the hazard of heart disease is better. Feature selection implementing improved the recital of process even more, and the results showed a significant increase in accuracy prediction. For weak classifiers, using ensemble methods resulted in a maximum increase in accuracy of 7%. ML algorithms have gained admiration in recent years owed to their increased accuracy as well as efficiency in predictions [19].

The capacity to generate and indicate models thru maximum accuracy and efficiency is critical in this field [20]. Because they mix several ML models with data systems, hybrid models [21] are a viable approach to illness prediction. The accuracy of weedy classifiers was enhanced through bagging besides boosting approaches, and the concert for risk detection of heart disease good rated. For the hybrid model development, they employed Bayes Net, NB, C 4.5, MLP, and, RF classifiers with majority voting. The created model has an accuracy of 85.48%. The UCI heart disease dataset has recently been subjected to ML techniques such as RF, SVM, besides learning models [22]. The voting-based model improved accuracy used in conjunction through multiple classifiers. Rendering to the research, the anemic classifiers improved accuracy by 2.1%.

ML classification techniques to forecast chronic illness were used in [23]. The Hoeffding classifier correctly predicted heart disease with an accuracy of 88.56% in their study. According to their findings, when collective with the specified characteristics, the hybrid model achieved an accuracy of 87.41%. The SVM classification model was used with the mean feature selection Fisher score strategy in [24]. In [25], the authors developed a unique prediction model based on many well-known classification approaches and a range of feature combinations. In the suggested HRFLM, an ANN with back propagation in addition 13 clinical characteristics as inputs was employed, and data mining approaches such as DT, SVM, NN, and KNN were explored. SVM has been shown to improve illness prediction accuracy. A new technique, vote, was presented, as well as a hybrid strategy combining LR and NB. Using the HRFLM method, an accuracy of 88.7% was achieved. An inclusive risk approaches for predicting heart fiasco mortality was constructed using an improved RandomSurvivalForest(iRSF) with great accuracy [26]. Using a unique split rule and stop criteria, iRSF was able to discriminate among survivors as well as non-survivors. A data mining method has also been used to diagnose cardiovascular disease [27].

To diagnose cardiac disorders, Bayesian, DT classifiers, NN, Association Law, KNN, and SVM, ML algorithms remained utilized. The accuracy of the SVM was 99.3%. Patient survival has been predicted using many machine learning classifiers [28]. Traditional biostatistics tests were compared to the offered ML methods, and characteristics associated with significant risk factors were graded. As a consequence, serum creatinine then ejection fraction was revealed to be the two utmost critical elements in generating accurate predictions. The ML algorithm [29] was used to build a CVD detection model. The dataset was prepared and investigated using four algorithms. The DT and RF methods had a precision of 99.83%, while the SVM and KNN methods had a precision of 85.32% and 84.49%, respectively. Another study [30] used the ensemble method to predict congestive heart failure (CHF) by analyzing heart rate variability (HRV) and filling in the gaps in related fields using deep neural networks. The proposed system’s accuracy rate was 99.85%.

In a recent paper [31], the authors developed an intelligence framework using mixed data factor analysis and RF-based MLA. RF was used to predict disease by means of the FAMD to treasure the applicable features. The precision of the proposed system was 93.44%, the sensitivity was 89.28%, and the specificity was 96.96%. In [32], the authors used a dataset with 303 instances, which was derived from the Cleveland dataset, to test their hypothesis. The proposed algorithm DT achieved a 75.55% accuracy rate.

Heart disease is frequently recognized as a cardiovascular disease. Several investigators are working on the forecast of heart disease. Their studies cover many aspects of cardiac illness. In [33], the author applied the REP Tree, R Tree, M5P Tree, LR, J48, NB, and JRIP on Hungarian and Statlog datasets to classify CVD. RF, DT, and LR are applied in [34]. AB, ET, LR, MNB, SVM, CART, LDA, XGB, and RF are applied in [35]. The purpose of this research is to predict the probability of people getting heart illness. The findings of [34] elaborates that LR reaches 92% accuracy, and in [35], SVM performs better by achieving 96% accuracy. In [36], the author claims that the DT model consistently beats the NB and SVM models. Its results show that SVM achieves 87% accuracy and DT achieves 90% accuracy, as shown in [37], while LR achieves the maximum accuracy in the prediction of heart disease at what time when equated to DT, SVM, NB, and KNN. The prediction accuracy provided by the RF-based framework is 97% [38], with a specificity of 88% and a sensitivity of 85% for the evaluation of congenital heart disease. In [39], we applied LR, MARS, EVF, and CART-ML techniques to perceive the co-existence of CVD and 94% accuracy, with a specificity of 95% and sensitivity of 93.5%. RF was applied in [40] for the prediction of medication targets involved in microorganism-associated CVD of host–host interactions and host–pathogen interactions.

To achieve a better solution, researchers proposed several ensembles and hybrid representations for cardio disease prediction. The proposed technique in [41] achieve 96%, 88.24% and 93%, accuracy on CVD obtained from the Mendeley Center, Cleveland datasets, and IEEE Port respectively. In [42], the author hybridized the LR and RF models for predicting heart disease and achieved an 88.7% accuracy level. These studies aim to investigate relatives between coronary artery calcium and carotid plaque in a-symptomatic entities, likewise in relative to predicted CVD occurrence risk [43]. Machine learning techniques combined with the IoT are currently widely used for predicting and detecting diseases. In [44], using mobile device technology, the author applied the deep learning approach and achieved a 94% accuracy in heart illness prediction. In [45], the author conjuncts the IoT with ML classifiers for the early prediction of heart infections. The objective was to demonstrate how ML may be used to solve the problem. We use ML to analyze cases associated with diseases and health conditions by analyzing hundreds of healthcare datasets [46].

In [47], the researchers worked on the advanced computer Vision for dependable Healthcare to determine how the computer vision practices support human needs such as psychological functioning, particular mobility, sensory functions, regular living activities, image processing, machine learning, pattern recognition, and how language processing then computer graphics collaborate with robotics. The authors observed and described how the users learn about emergent computer vision techniques for assisting mental functioning, approaches for investigating human behavior, and how keen interfaces and virtual realism tools contribute to the development of advanced restoration systems capable of performing human actions and activity recognition. The works support the existing contribution of computer vision in the health care sector such as the technologies behind the intelligent wheelchairs, potential help for blind people, and other computer vision-based solutions that have recently been used for safety and health monitoring. In [48], the authors applied multiple approaches such as SVM, GNB, LR, LightGBM, XGB, and RF for envisaging the heart disease risk. RF performed the best, achieving 88% accuracy for foreseeing the heart disease. The latest work of researchers is compared with our proposed approach. Our proposed approach achieves the highest accuracy as compared to the existing approaches that use the UCI repository dataset. Along with this, we evaluated accuracy, precision (specificity), recall (sensitivity), and F-Measure using the ten ML classifiers.

The etiology of cardiac disease is tranquil an unresolved global problem, and the main characteristics of cardiovascular diseases are high morbidity, disability, and mortality. As a result, efficient and effective early forecast of the likely results in affected role with cardio disease with AI is required. In this study, we applied an ensemble ML model for coronary disease prediction. In this work, ML classifiers are used to predict cardiac disease. The authors begin by addressing the dataset issue, which they then refine and standardize for tokenization and lower casting. Afterwards, the datasets were used to train and test the classifiers to evaluate the performance and to achieve the optimum accuracy. The inclusion criteria of these algorithms are to be state-of-the-art and representative and have high maturity. By analyzing the earlier researchers’ works, we used the Gradient Boosted Tree (GBT) and Multilayer Perceptron (MLP) earlier. We analyzed that the previous researchers had not used them on UCI heart dataset.

The significant contributions of the future effort are as follows:

(1) Firstly, Authors begin by addressing the matter of datasets, which they then refine and standardize. The datasets are then castoff to train in addition test classifiers in order to determine which ones provide the finest accuracy.
(2) Secondly, authors categorize the best values or features using the correlation matrix.
(3) Thirdly, the authors applied the ML classifiers to the preprocessed dataset to obtain the maximum accuracy which was performed through parameter tuning.
(4) Fourthly, the proposed classifiers are evaluated on accuracy, precision (specificity), recall (sensitivity), and F-Measure.
(5) Finally, the proposed classifiers give better accuracy as associated to the accuracy of state-of-the-art as listed in Table 1.

In this work, the prediction accuracy of several ML approaches is investigated to evaluate coronary heart disease. The investigation of several ML classification approaches was performed on well-known UCI repository heart disease datasets using the following hardware and software: Processor Intel (R) Core (TM) i5-8256U CPU @ 1.602GHZ (8CPUs) 1.8 GHz, Memory 8192 MB RAM, Software Python, Jupyter Notebook

The comparison of the performance of the latest Gradient Boosted Tree, Multilayer Perceptron, and Random Forest along with these seven other ML classifiers in terms of cardiovascular disease prediction is inimitable. As a result, a system for predicting heart problems that are both efficient and accurate is now accessible. Furthermore, we endorse the best-suited ML classifier for designing and developing high-level intelligent systems to predict coronary heart disease.

## 3. Proposed Methodology

We used the ML classifiers to predict the existence of coronary heart disease with the heart dataset. The dataset was retrieved as of the UCI-repository [49], and data pretreatment was performed before selecting the features using feature engineering. Then, we fragmented it into two parts: a training dataset and a test dataset; around 70% of the entire data is utilized for training, whereas the rest is used for testing. The test dataset is utilized to assess classifiers, while the training is to develop a model that predicts heart disease. First, we explore the dataset before converting categorical values to numerical values for categorization.

**Figure 1:**
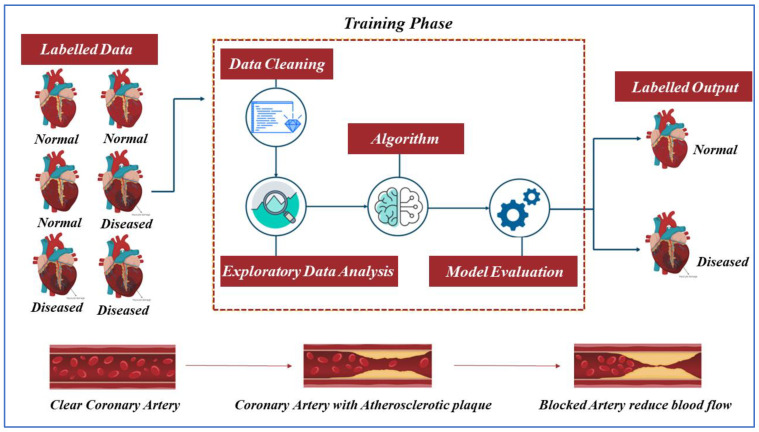
System Working Methodology.

In Step 1, we labeled the dataset with the “normal” and “diseased” labels. The normal label represents that a person is free from heart disease, and the diseased label shows that a patient is facing a heart problem. Then, in the training phases, in Step 2, we performed the data cleansing. As the dataset contains missing and incomplete values, we performed the data preprocessing and filled in the missing values by taking the mean. In Step 3, we performed the data visualization using the Exploratory Data Analysis (EDA) (discussed in Section 4) to check the correlation between different attributes. We noticed that FBS has a very weak correlation. After this, in Step 4, we applied the ML classifiers to the preprocessed dataset and evaluated the performance of the classifiers on different parameters. As discussed above, the dataset is split into test and training sets to evaluate the classifiers and train the model, respectively. The applied classifiers show different accuracies aimed at predicting the presence of heart illness. The phases of our proposed working technique are depicted in Figure 1.

The coronary artery contour shows the conditions of the coronary artery, such as the clear coronary artery (artery before the heart problem), the artery with atherosclerotic plaque, and the blocked artery that reduces the flow of the blood.

### 3.1. Dataset

The heart disease datasets were taken from the UCI repository [49]. This dataset comprises 303 instances, multivariate characteristics, containing the integer, categorical, and real values, and 14 attributes. The dataset’s description is provided in Table 2.

### 3.2. Correlation Matrix

Correlation is a statistical feature that describes the strength and route of a linear relationship among two quantitative variables. The correlation between the columns is labeled in Table 3. The majority of columns have a moderate correlation with the “num” variable, but ‘FBS’ has a very weak correlation.

**Figure 2:**
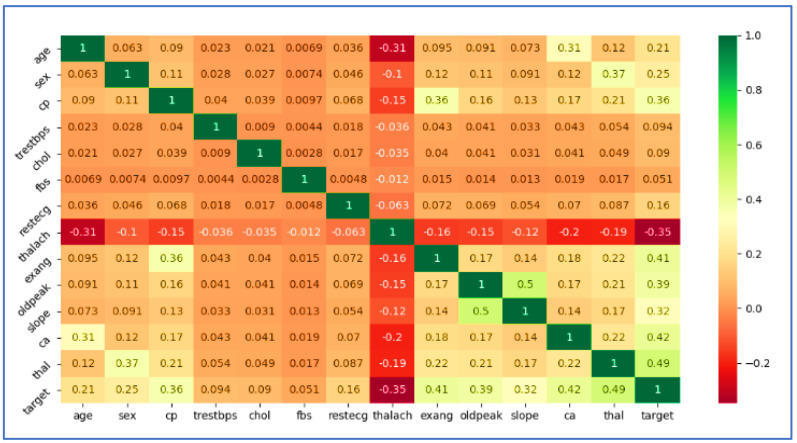
Correlation Matrix with a Heatmap.

A correlation matrix with heatmap is shown in Figure 2. Using a heatmap, you can see how dependent values are affected by independent features. Furthermore, it is easy to see which features are greatest associated with the additional features variable. Figure 2 depicts the results.

## 4. Result and Analysis

In this Section, we plot the feature of the heart disease dataset vs. num (predictive attribute) for data visualization. Exploratory Data Analysis (EDA) is a technique used for analyzing datasets to summarize their main characteristics, which is frequently accomplished through the use of statistical graphics and other data visualization methods.

### 4.1. Disease Status

**Figure 3:**
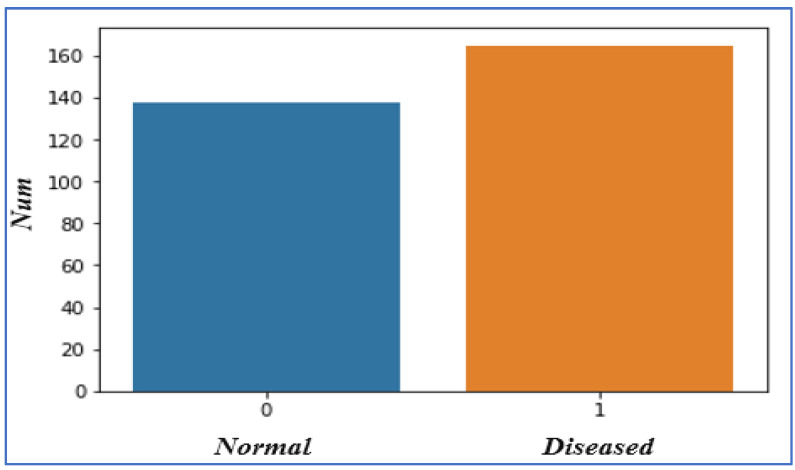
Heart Disease Status.

In diseased states, we concluded that, from a total of 303 instances, 165 patients had a heart disease problem. We represent ‘diseased’ with 1 and ‘normal’ with 0, and 138 patients are normal out of the total instances. From this, we derived that the percentage of patients who face heart glitches is 54.46%, and the fraction of patients without heart problems is 45.54%, as shown in Figure 3. We also analyzed the other dataset attributes such as Age, Chest Pain, Sex, Exercise-Induced Angina, Fasting Blood Sugar, Resting ECG, Slope, Coronary Artery, and Thalassemia features.

### 4.2. Analyzing Sex

**Figure 4:**
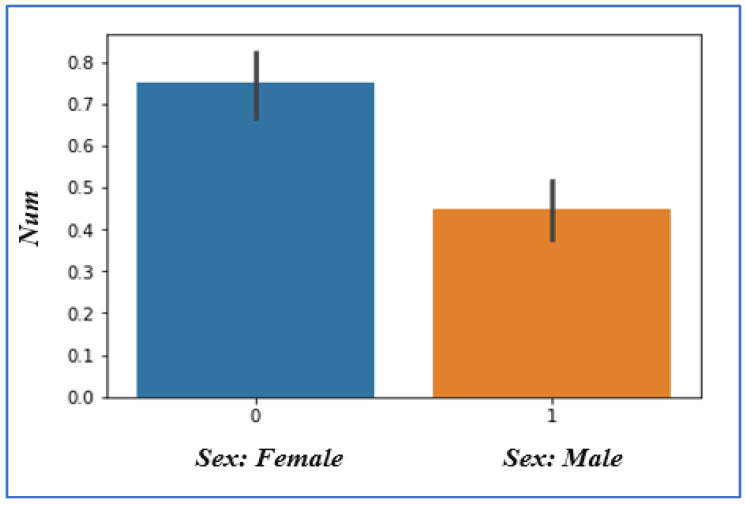
Sex heart disease chances.

In the sex attribute, we have two values, male and female: 0 is used for females, and 1 is used for males, as shown in Figure 4. Females are additional likely to have heart problems than males, according to the findings.

### 4.3. Analyzing Age

**Figure 5:**
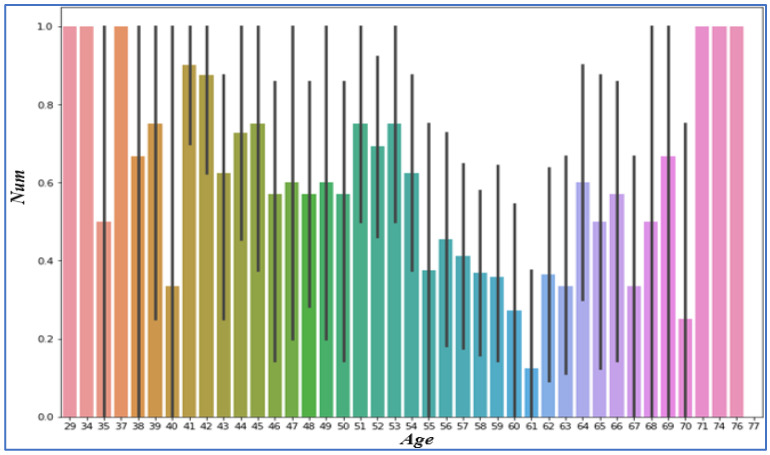
Heart Disease Dataset Age Statistics.

We can see in the Figure below that the chances of heart disease do not depend upon age, as shown in Figure 5 dataset age statistics. The x-axis signifies age, while the y-axis epitomizes the target percentage.

### 4.4. Analyzing Chest Pain

**Figure 6:**
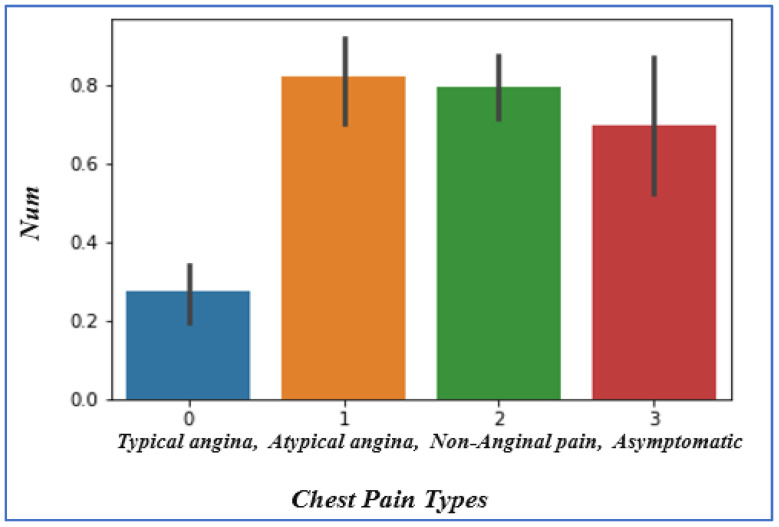
Chest pain vs. heart disease chances.

Patients with heart disease may experience chest pain. As shown in Figure 6, we looked at chest pain in the subsequent categories: non-anginal pain = 2, asymptomatic = 3, atypical angina = 1, typical angina = 0. We have noticed that people who have ‘0’ chest pain, i.e., those who have typical angina, are considerably less likely to have heart difficulties. Patients who have atypical angina have increased chances of heart disease occurrence.

### 4.5. Analyzing Fasting Blood Sugar

**Figure 7:**
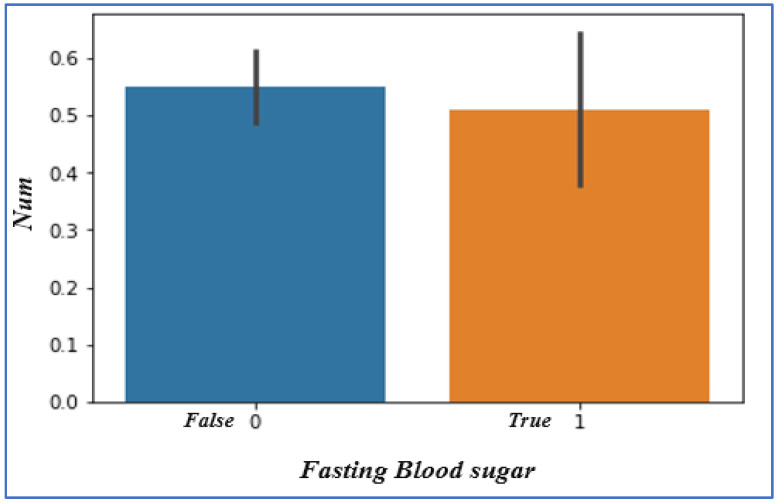
Fasting blood sugar vs. disease chances.

Fasting blood sugar (FBS) cannot play many roles in heart disease occurrence. We analyzed the dataset in which if the patient’s fasting blood sugar level exceeds 120 mg/dL, it means that they are facing it, and we represent it by the value 1 (True); the other case is represented by the value 0 (False), as shown in Figure 7. The outcome shows that there is nothing extraordinary here for predicting the presence of heart disease.

### 4.6. Analyzing Resting ElectroCardioGraphic

**Figure 8:**
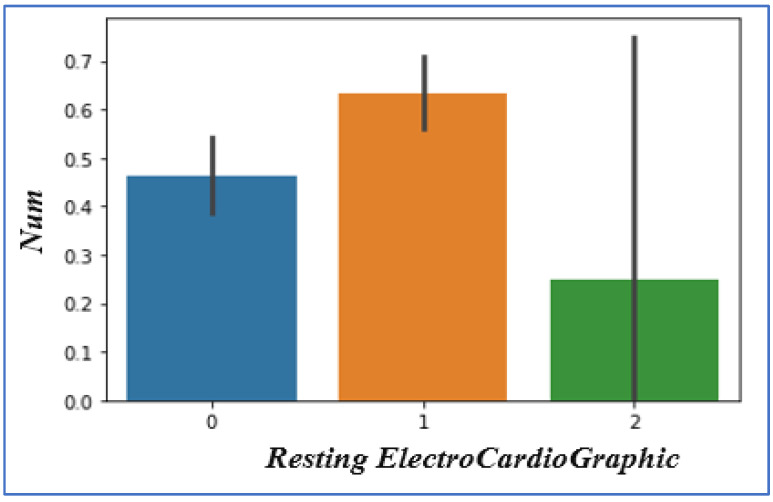
Resting ECG vs. heart disease chances.

Resting ElectroCardioGraphic values are 0, 1, and 2. The outcome shows that individuals with Resting ECG values of ‘1’ and ‘0’ have increased chances of heart disease as compared to Resting ECG value ‘2′, as presented in Figure 8.

### 4.7. Analyzing Exercise-Induced Angina

**Figure 9:**
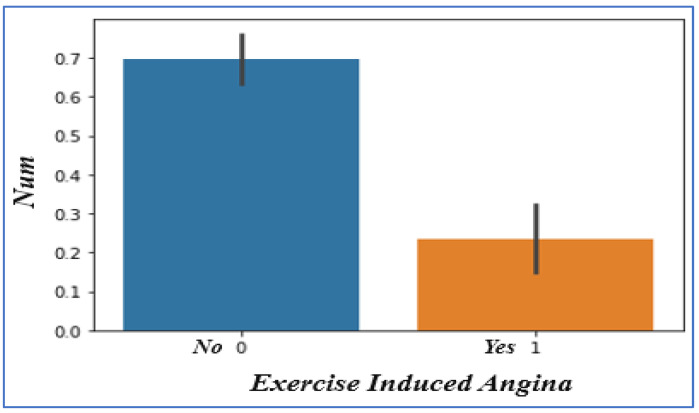
Exercise-Induced Angina vs. disease chances.

In Figure 9, people with angina are considerably less likely to have heart problems. If the value of exercise-induced angina is 1, it means ‘yes’, the patient has a heart problem; if it is 0, it means ‘no’, the patient is less likely to have heart problems.

### 4.8. Analyzing Slope

**Figure 10:**
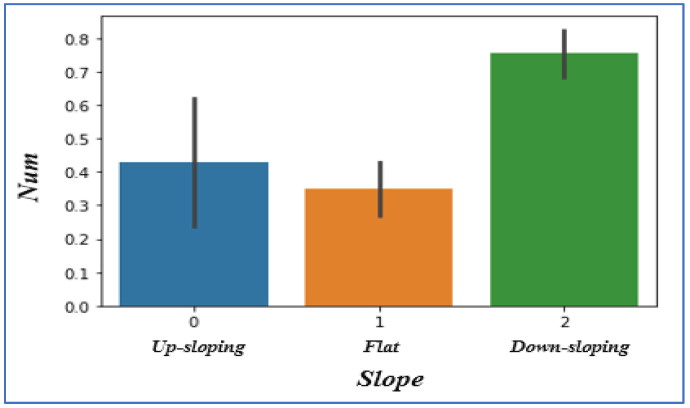
Slope vs. heart disease chances.

We have three different types of slopes that cause heart problems: upsloping, downsloping, and flat. After visualizing the data, we notice the Slope ‘2’ cause significantly more heart pain Slope ‘1’ or Slope ‘0’, as shown in Figure 10.

### 4.9. Analyzing Coronary Artery

**Figure 11:**
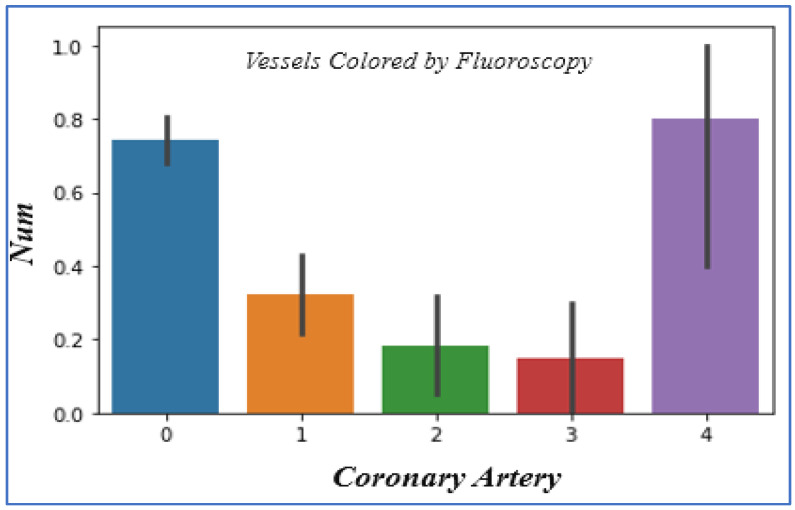
Coronary Artery vs. disease chances.

In analyzing the coronary artery attribute of the heart disease dataset, we obtain the value of main vessels tinted by fluoroscopy, and its value is 0–4. If the value of the coronary artery is 4, there is an astonishingly great number of patients facing heart problems, as shown in Figure 11.

### 4.10. Analyzing Thalassemia Affects the Heart

**Figure 12:**
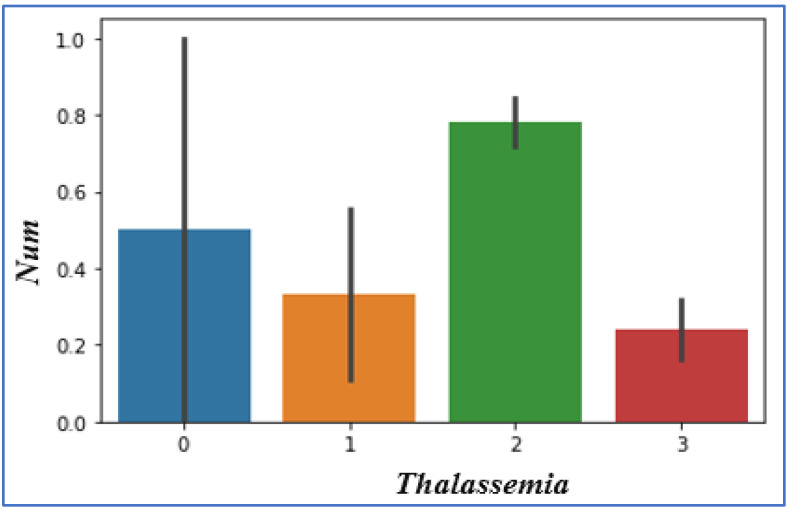
Thalassemia vs. heart disease chances.

Thalassemia affects the heart. In this heart disease dataset, we can get the value of thalassemia as normal, fixed defect, and reversible defect. The values given in the dataset are 0, 1, 2, and 3, as shown in Figure 12. From these values, it is detected that if the value of thalassemia is 2, it means the patient has a higher chance of carrying the heart disease problem.

## 5. Result and Discussions

In this Section, the outcomes of ML classifiers on different evaluation constraints such as precision, recall, and F-measure are discussed. Along with this, the accuracy of machine learning classifiers on the heart disease dataset is evaluated. kNN did not perform well; however, the RF, GBT, and MLP performed better as compared to other classifiers.

### 5.1. Evaluating Parameters

Accuracy, recall, precision, and F-measure are the main evaluation parameters considered in this research to evaluate the ML classifier’s performance, as presented in Table 4. Consequently, the specificity (precision) and sensitivity (recall) of the focused class are computed to inspect the predicted accuracy of the particular algorithm. The accuracy, precision, recall, and F measure in ML are calculated using the “*TP*—True Positive, *TN*—True Negative, *FN*—False Negative and *FP*—False Positive,” rate. All true positive and true negative predictions are split into all positive and negative predictions. All models predicted *TP*, *TN*, *FN*, and *FP*. Diseased is denoted by the letters *TP*. *FN* is a disease that is anticipated to not be heart disease. *FP* is a disease that was anticipated but never manifested. *TN* is not a disease in the real world, and it is not expected to be one in the future.

- *Accuracy* is measured as the number of fittingly identified examples divided by the total occurrences in the dataset as in Equation (1).

(1) $$Accuracy=\frac{TP+TN}{TP+FP+TN+FN}\;\;*\;100\;$$

- *Precision*: the average likelihood of retrieving relevant information, as indicated in Equation (2).

(2) $$Precision=\frac{TP}{TP+FP}\;\;$$

- Recall: the average likelihood of complete retrieval, which is defined in Equation (3).

(3) $$\mathrm{Recall}\;=\frac{TP}{TP+FN}\;\;\;$$

- F-Measure: once the *precision* and recall for the classification problem have been calculated, the two scores are combined to compute the F-Measure. The conventional F measure is computed as shown in Equation (4).

(4) $$\mathrm{F-Measure}=\frac{\left(2*Precision*Recall\right)}{Precision+Recall}\;\;\;$$

### 5.2. Performance of ML Classifiers

**Figure 13:**
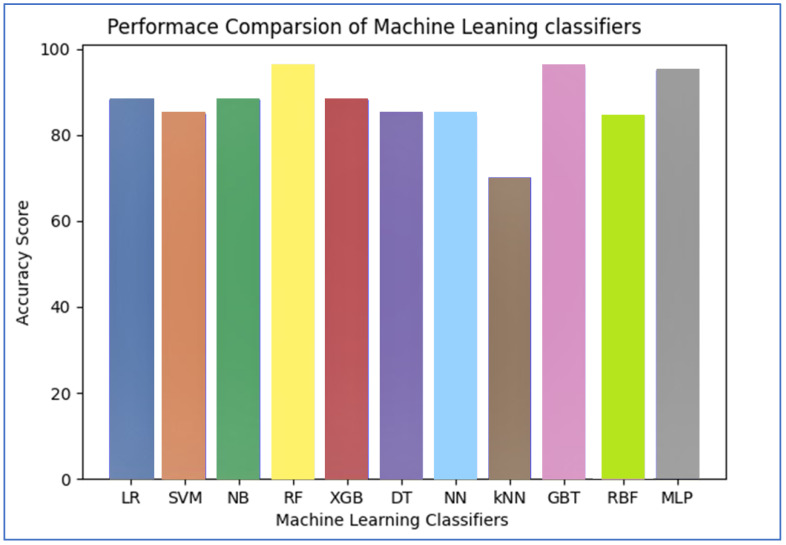
ML Classifiers Accuracy.

The performance of ML approaches in terms of accuracy is listed in Table 4. By associating the performance of these classifiers, we observed that Random Forest, Gradient Boosting Tree, and Multilayer perceptron performed well as, related to other ML classifiers, these models attained almost 96.28%, 95.83%, and 95% accuracy respectively, as shown in Figure 13.

**Figure 14:**
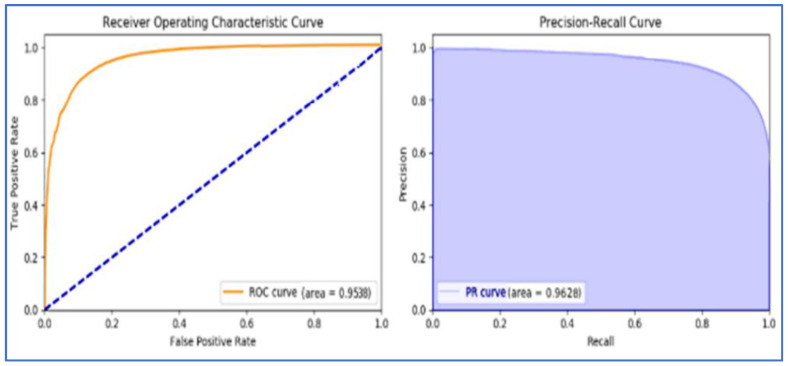
Random Forest Classifiers ROC.

**Figure 15:**
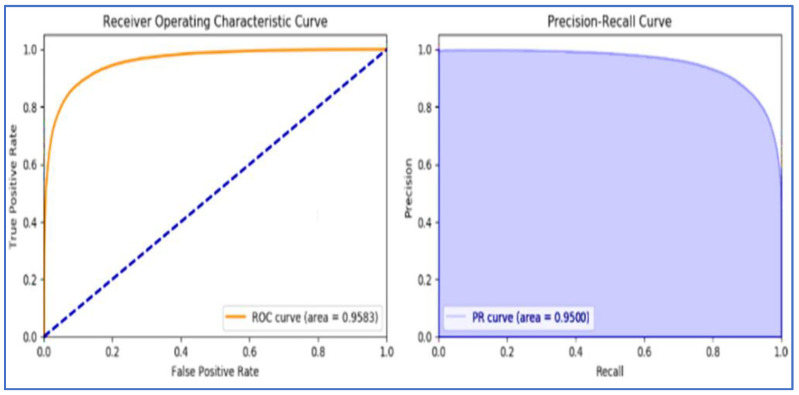
Gradient Boosting Tree Classifiers ROC.

**Figure 16:**
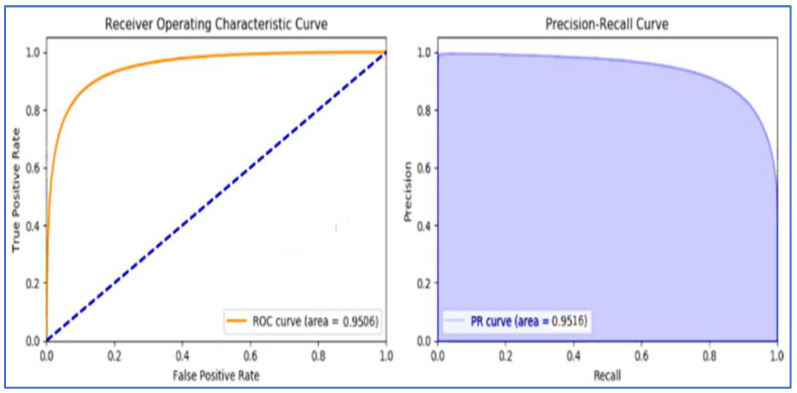
Multilayer perceptron Classifiers ROC.

The ROCs (Receiver Operating Characteristic Curves) of these effective techniques such as RF, GBT, and MLP are represented in Figure 14, Figure 15, and Figure 16, respectively.

## 6. Conclusions and Future Work

In this paper, ML classifiers are used to predict the presence of heart problems. The dataset was attained from UCI repository. The gained data is cleansed, and preprocessing is performed. After that, ML models are applied for predicting. The potential of these eleven applied ML approaches for predicting cardiac disease was assessed. The inclusion criteria of these algorithms are to be state-of-the-art and representative and have high maturity. By comparing with existing work, we have used the Gradient Boosted Tree (GBT) and Multilayer Perceptron (MLP) earlier, but other researchers have not used them on the UCI heart disease dataset, and we have achieved more accuracy compared to them, as described in the ‘state of the art’ table. The resultant outcomes reveal that from the applied ML classifiers, the Gradient Boosted Tree and Multilayer Perceptron achieve 95% accuracy in predicting the presence of coronary heart disease. However, the highest classification accuracy of 96.28% was achieved using Random Forest (RF) with a specificity and sensitivity of 0.9628 and 0.9537, respectively.

In the future, we will use the additional datasets to try to obtain more reliable conclusions, and we will optimize the parameters of the ML classifiers and deep learning methods using metaheuristic techniques and nature-inspired algorithms to more effectively evaluate the presence of heart disease through different heart disease-related datasets, as well as trying to enhance the accuracy of the existing algorithms.

## Figures and Tables

**Table 1:** State of the Art.

Author	Year	Methods/Classifiers	Datasets	Evaluation Parameters	Highest Accuracy%
[36]	2022	LR, NB, RF REP, M5P Tree, J48, JRIP	Hungarian and Statlog (heart) dataset	RMSE, MAE	RF 99.81%
[37]	2021	RF, DT, LR	UCI Cleveland database	Accuracy	LR 92.10%
[38]	2021	AB, ET, LR, MNB, CART, LDA, SVM, RF, XGB	Heart Dataset (UCI repository)	Accuracy	AB 90%
[39]	2021	SVM, NB, DT	Heart Dataset (UCI repository)	Accuracy	DT 90%
[40]	2022	KNN, DT, LR, NB, SVM	Heart Dataset (UCI repository)	Accuracy, Specificity, Sensitivity, F1-Score	LR 92%
[41]	2022	RF into fetal echocardiography	Congenital heart disease database of 3910 Singleton Fetuses	Sensitivity, Specificity	sensitivity 0.85, specificity 0.88,
[42]	2022	LR, Evimp functions, Multivariate adaptive regression	DiScRi dataset	Accuracy, Sensitivity, Specificity	94.09%
[43]	2022	LR, KNN, SVM, RF	Pathogen, Host feature	Accuracy	RF 99%
[44]	2022	DT, LR, XGB, NB, GB, RF, SVM, PEM	Cardiovascular disease dataset (Mendeley Data Center)	Accuracy	EM 96.75%
[45]	2021	NB, LM, LR, DT, RF, SVM, HRFLM	Heart Cleveland (UCI repository)	Accuracy, Precision, Specificity, Sensitivity, F-Measure	HRFLM 88.4%
[47]	2021	RF, LR, KNN, SVM, DT, XGB	Public Health Dataset	Accuracy, Specificity, Sensitivity	SVM 84%
[48]	2022	K-NN, DT, RF, MLP, NB, L-SVM,	IoT based Produced Data	Accuracy	L-SVM 92.30%, RF 92.30%
[49]	2022	DT, NB, KNN, RF, ANN, Ada, GBA	Heart Disease (Kaggle Repository)	Accuracy, Precision, recall, f1-score	RF 86.89%
In our Proposed Scheme					
Proposed Methodology	2022	LR, SVM, NB, RF, XGB, DT, NN, RBF, KNN, GBT, MLP	Heart Disease (UCI Repository)	Accuracy, Precision (specificity), Recall (sensitivity), F-Measure	RF 96.28%

**Table 2:** Dataset Attributes Description.

Dataset Details			
No.	Features	Description	Value
1.	Age	Age is an important aspect of health care.	Its value is an integer.
2	Sex	Gender	Female = 0, Male = 1
3.	Chest pain(cp)	The patient is suffering from chest pain.	Asymptomatic = 4, typicalangina = 1, atypicalangina = 2, non-anginal pain = 3
4.	RestingBloodPressure (trestbps)	High blood pressure ensues with some other factors which increase the risk.	It has either an integer or float value.
5.	Cholesterol(Chol)	Serum cholesterol	It has either an integer or float value
6.	FastingBloodSugar(Fbs)	Fasting blood sugar is more than 120 mg/dL	0 = false; 1 = true
7.	RestingECG (restech)	ElectroCardioGraphic Resting	ST-T wave abnormality =2, Normal =0, Left ventricular hypertrophy =1,
8.	Max Heart Rate Achieved (thalach)	This is the highest heart rate you have ever had.	It has either an integer or float value.
9.	Exercise-Induced Angina (exang)	Angina instigated by exercise	no = 0, yes = 1
10.	Oldpeak	Exercise-tempted ST depression compared to rest	It shows the value as either an integer or a float.
11.	Slope	slope of peak exercise ST segment	flat = 1, downsloping = 2, Upsloping =0
12.	Coronary Artery (ca)	Fluoroscopy has colored a large number of major vessels.	It has either an integer or float value.
13.	Thalassemia (thal)	Normal, reversible defect, fixed defect,	Measuring scales: 3 = normal; 7 = reversable defect; 6 = fixed defect
14.	Num(target: Heart Disease predicting attribute)	Heart disease diagnosis (angiographic disease status)	0 indicates a diameter narrowing of less than 50%, 1 indicates a diameter narrowing of more than 50%.

**Table 3:** Correlation Matrix Value.

Attributes	Value
Age	0.225439
Sex	0.280937
Chest Pain	0.433798
Fasting Blood Sugar	0.028046
Resting Blood Pressure	0.144931
Cholesterol	0.085239
Exercise-Induced Angina	0.436757
Max Heart Rate Achieved	0.421741
Resting ECG	0.137230
Oldpeak	0.430696
Slope	0.345877
Coronary Artery	0.391724
Thalassemia	0.344029
Heart Disease Diagnosis	1.000000

**Table 4:** Accuracy of ML Classifiers.

Classifiers	Accuracy	Precision	Recall	F-Measure
Logistic Regression	88.25%	0.8791	0.8825	0.8865
Support Vector Regression	84.97%	0.8407	0.8496	0.8437
Naive Bayes	88.25%	0.8825	0.8854	0.8825
Random Forest	96.28%	0.9628	0.9537	0.9668
XGBoost	88.25%	0.8786	0.8810	0.8815
Decision Tree	84.97%	0.8497	0.8475	0.8527
Neural Network	84.33%	0.8433	0.8501	0.8413
k-Nearest Neighbors	70.21%	0.7021	0.6901	0.7101
Gradient Boosted Tree	95.83%	0.9493	0.9583	0.9613
Radial Basis Function	86.35%	0.8635	0.8644	0.8635
Multilayer perceptron	94.96%	0.9516	0.9506	0.9506

## Data Availability

The data used in this research can be obtained from the corresponding authors upon request.

## Abbreviations

Notations	Description
ML	Machine Learning
UCI	University of California, Irvine
EDA	Exploratory Data Analysis
CVDs	CardioVascular Diseases
kNN	k-Nearest Neighbors
LR	Logistics Regression
RF	Random Forest
DT	Decision Tree
NB	Naïve Bayes
XGB	XGBoost
NN	Neural Network
ANN	Artificial Neural Network
SVM	Support Vector Machine
EVF	Evimp functions
CART	Classification and Regression
MARS	Multivariate Adaptive Regression Splines
FBS	Fasting Blood Sugar
Resting ECG	Resting ElectroCardioGraphic
PEM	Proposed Ensemble Model
